# Community-level Alzheimer’s Disease or Related Dementia’s 65+ Prevalence in Five New England States

**DOI:** 10.1093/geroni/igaf122.2848

**Published:** 2025-12-31

**Authors:** Taylor Jansen, Nina Silverstein, Yan-Jhu Su, Shan Qu, Mengshi Liu, Yan Lin, Qian Song, Elizabeth Dugan

**Affiliations:** University of Massachusetts Boston, Boston, Massachusetts, United States; University of Massachusetts Boston, Boston, Massachusetts, United States; The Pennsylvania State University, University Park, Pennsylvania, United States; University of Massachusetts Boston, Boston, Massachusetts, United States; University of Massachusetts Boston, Boston, Massachusetts, United States; University of Massachusetts Boston, Boston, Massachusetts, United States; University of Massachusetts Boston, Boston, Massachusetts, United States; University of Massachusetts Boston, Boston, Massachusetts, United States

## Abstract

An estimated two thirds of people living with Alzheimer’s disease or related dementias (ADRD) live and are cared for at home. For public health action and community programming to assist those living with ADRD, accurate disease rates must be known. The Healthy Aging Data Reports (HADR) (www.healthyagingdatareports.org) are a comprehensive tool to spur community and policy action as they calculate community-level prevalence of 38 chronic diseases for each community in a state. The present descriptive study will compare community and neighborhood-level 65+ prevalence of ADRD in five New England states: Connecticut (CT), Massachusetts (MA), Maine (ME), New Hampshire (NH), and Rhode Island (RI). Using Medicare Beneficiary Summary File (MBSF) (2020-2021), representative of 100% of traditional Medicare beneficiaries, small area estimation techniques were used to calculate age-sex adjusted community rates of ADRD and over 150 other health indicators. Results varied across states with NH reporting the lowest community rate of 65+ ADRD in the sample (5.41%) and CT reporting the highest (27.10%). CT and MA reported the largest range in 65+ ADRD (CT: 6.90-27.10%; MA: 6.69-25.54%) with the highest rate clusters located near the biggest cities, in urban areas (CT:13.11-27.10%; MA: 12.93-25.54%). Conversely, in Maine and NH, the highest rate clusters were found in rural areas (NH: 15.82-21.16%; ME: 15.33-18.98%). The 2025 HADR’s found geographic disparities in 65+ community-level ADRD across New England. Communities with high rates can be targeted for additional supports like caregiver support programming, adult day health centers, and educational programming on the prevention and treatment of ADRD.

